# Supplementary material for: High Platelet Reactivity after Transition from Cangrelor to Ticagrelor in Hypothermic Cardiac Arrest Survivors with ST-Segment Elevation Myocardial Infarction
Source: J Clin Med. 2020 Feb 21;9(2):583. doi: 10.3390/jcm9020583 (PMC7073541; doi:10.3390/jcm9020583)
Supplement: Supplementary file 1 [file jcm-09-00583-s001.pdf]

Supplement

Table S1: Results of Multivariable Poisson Regression

Exact Poisson regression

Number of observations = 16				
**hprtimepoi~s**	**IRR**	**Suff.**	**2*Pr(Suff.)**	**[95% Conf. Interval]**
timeticabe~g	9717907	365	0.0054	.950613 - .9918672
	1.0282093	365	0.0054	1.0081328 - 1.049387
timeticabe~g	.9685399	365	0.0043	.9458258 - .9903346
bnp	1.421757	10	0.5966	.5075964 - 3.884677
timeticabe~g	.9782004	365	0.0499	.9556211 - .9999894
asat	1.000424	20334	0.0775	.9999498 - 1.000877
timeticabe~g	.9749758	365	0.0265	.9517738 - .9971864
alat	1.000473	13881	0.1000	.9998996 - 1.001018
timeticabe~g	.9640086	365	0.0010	.9408023 - .9859071
ggt	1.016386	2400	0.0032	1.005273 - 1.02861
timeticabe~g	.9700878	365	0.0079	.9466079 - .99235
bili	.1565579	11.02	0.0419	.0206956 - .9437993
timeticabe~g	.975843	365	0.0155	.9548611 - .9955729
lvef	2.763996	13	0.0340	1.072052 - 7.618956
timeticabe~g	.9703227	365	0.0046	.9487045 - .9910101
heartrate	.9921897	1538	0.4986	.9699581 - 1.015488
timeticabe~g	.9720851	365	0.0060	.9507367 - .9922357
bpsys	.9971348	2461	0.7736	.978147 - 1.015937
timeticabe~g	.9755029	365	0.0180	.9534701 - .9959993
bpdia	.9847304	1385	0.3571	.9528364 - 1.017429
timeticabe~g	.9725562	365	0.0090	.9506217 - .993358
bpmean	.9959471	1769	0.7977	.965419 - 1.028282
timeticabe~g	.9693279	365	0.0030	.9477922 - .9898265
t	.6030332	728.6	0.1125	.3126586 - 1.118555
timeticabe~g	.9754559	365	0.0149	.9540476 - .9953878
noradrenalin	8.92275	3.376	0.1091	.5845038 - 111.3444
timeticabe~g	.9905771	365	0.4603	.9655298 - 1.014458
rocuronium	.8825178	389	0.0167	.7824801 - .979794
timeticabe~g	.9667655	365	0.0014	.9474538 - .9865202
insulin	1.311264	14	0.1349	.9060454 - 1.809148
timeticabe~g	.9721758	365	0.0056	.9511526 - .9920569
remifentanil	1.448134	19	0.8040	.4227409 - 7.927062
timeticabe~g	.9751322	365	0.0262	.9526267 - .9970661
dobutamin	1.135581	17.76	0.5162	.811387 - 1.511731
timeticabe~g	.9663937	365	0.0034	.941191 - .9893308
levosimendan	44752	.75	0.0142	9.535629 - 7.66e+07
timeticabe~g	.9786942	365	0.0614	.9559371 - 1.001019
amoxicillin	3.469221	9	0.0160	1.251505 - 9.323602
timeticabe~g	.9718895	365	0.0089	.9500632 - .9930202
amiodaron	1.013099	6	1.0000	.3119419 - 2.884679

We used exact Poisson regression to estimate the effect of the overlap-time of ticagrelor and cangrelor co-administration in minutes and potential confounding co-variables on the number of subsequent HPR episodes, expressed as incidence rate ratio (IRR) with a 95% confidence interval (95%CI). We used regular Poisson regression to estimate the constant. Confounding co-variables included co-administered drugs, blood pressure levels, heart rate, left ventricular systolic function as well as pro-BNP, liver enzyme and bilirubin levels at the time of transition from cangrelor to ticagrelor. There was a significant relationship between the overlap-time of ticagrelor and cangrelor co-administration and the number of subsequent HPR episodes with an IRR of 1.03, 95%CI 1.01-1.05; p=0.005. The effect remained unchanged after adjustment for co-variables.
